# Improvement of Functional Mobility Using a Hip-Wearable Exoskeleton Robot in Guillain-Barré Syndrome With Residual Gait Disturbance: A Case Report

**DOI:** 10.7759/cureus.63882

**Published:** 2024-07-05

**Authors:** Jun Yabuki, Kenichi Yoshikawa, Kazunori Koseki, Kiyoshige Ishibashi, Akira Matsushita, Yutaka Kohno

**Affiliations:** 1 Department of Physical Therapy, Mejiro University, Saitama, JPN; 2 Department of Physical Therapy, Ibaraki Prefectural University of Health Sciences Hospital, Ami, JPN; 3 Department of Neurosurgery, Ibaraki Prefectural University of Health Sciences Hospital, Ami, JPN; 4 Department of Neurology, Ibaraki Prefectural University of Health Sciences Hospital, Ami, JPN

**Keywords:** guillain–barré syndrome, rehabilitation, physical therapy, robotic-assisted gait training, walking ability

## Abstract

Patients with Guillain-Barré syndrome (GBS) occasionally have residual gait disturbance one year after disease onset. We hypothesized that providing hip joint movement assistance can improve gait in patients with GBS and residual gait disturbance. A 78-year-old man with GBS showed improvement in gait following conventional rehabilitation and gait training using GAIT TRAINER HWA-01 (HWA-01; Honda Motor Co., Ltd., Tokyo, Japan), which is a hip-wearable exoskeleton robot. Initially, he presented with gastrointestinal symptoms, subsequently flaccid quadriplegia, and respiratory muscle paralysis. He was diagnosed with acute motor axonal neuropathy and was transferred to our hospital on day 185 after the disease onset. Seven months after rehabilitation, his walking ability plateaued. On day 382, a single-case study with ABABA design intervention, with conventional gait training in phase A and gait training using HWA-01 in phase B, was conducted. The primary outcomes included a comfortable walking speed, stride length, and cadence. Comfortable walking speed, stride length, and cadence statistically improved after gait training using HWA-01. Furthermore, improvement in exercise capacity and activities of daily living exceeded the minimal clinically important difference for the intervention. The use of the HWA-01 gait trainer potentially improves gait in patients with GBS who have residual gait disturbance.

## Introduction

Guillain-Barré syndrome (GBS) is a rapidly progressive immune peripheral neuropathy caused by the transient induction of an aberrant autoimmune response to peripheral nerve tissues, due to stimuli, such as upper respiratory tract infections or gastroenteritis [1,2]. In patients with GBS, symptom progression peaks at two to four weeks after disease onset and then plateaus, after which the recovery of function may last months to years [3]. In particular, fatigue is a major symptom in patients with GBS and persists even after functional recovery [4]. In a systematic review of rehabilitation interventions for GBS, four studies involving 207 patients reported that gait training, exercises that can prevent and improve limitations in range of motion (ROM) and muscle weakness, and high-intensity rehabilitation programs are effective for enhancing the performance of activities of daily living (ADL) in patients with GBS [5]. However, some patients with GBS cannot walk independently or require walking aids one year after disease onset [6]. Moreover, the gait patterns of patients with GBS have been reported to show asymmetry and narrow hip, knee, and ankle joint angles compared with healthy adults [7].

The recently developed hip-wearable exoskeleton robot, GAIT TRAINER HWA-01 (HWA-01; Honda Motor Co., Ltd., Tokyo, Japan), can detect the patient’s gait rhythm, and effectively provide hip flexion and extension assistance [8]. The HWA-01 can be used by a single physiotherapist and it facilitates gait training using an algorithm wherein an angle and torque sensor located in the hip joint monitors the angle and regulates gait. Previous trials including patients with spinal cord injuries, orthopedic disorders, and stroke found that gait training with HWA-01 (HGT) effectively provided sufficient exercise intensity, promoted coordinated movement during gait, and increased stride length [8-11]. However, limited studies have been conducted on the effect of robot-assisted gait training on patients with GBS [12]. In particular, no study has examined the effect of HGT on patients with GBS. Therefore, a gait training program for patients with GBS that can prevent excessive fatigue and improve gait ability must be provided. We hypothesized that HGT can improve gait disturbance in patients with GBS. Herein, we present a case of a patient with GBS who showed improvement in HGT.

## Case presentation

Case description

A 78-year-old man (height: 153 cm, weight: 43.5 kg) was diagnosed with GBS (acute motor axonal neuropathy) based on positive anti-GM1 and anti-GQ1b antibodies. He presented with flaccid quadriplegia and respiratory paralysis induced by previous gastrointestinal symptoms. On day 185 after the disease onset, he was transferred to our hospital and started rehabilitation programs. The rehabilitation programs included physical therapy involving basic exercises, such as turning over, self-lifting, standing-up activities, and body weight-supported treadmill training; occupational therapy involving upper limb training and ADL training (eating, glooming, and dressing); and speech therapy involving dysarthria training.

**Figure 1:**
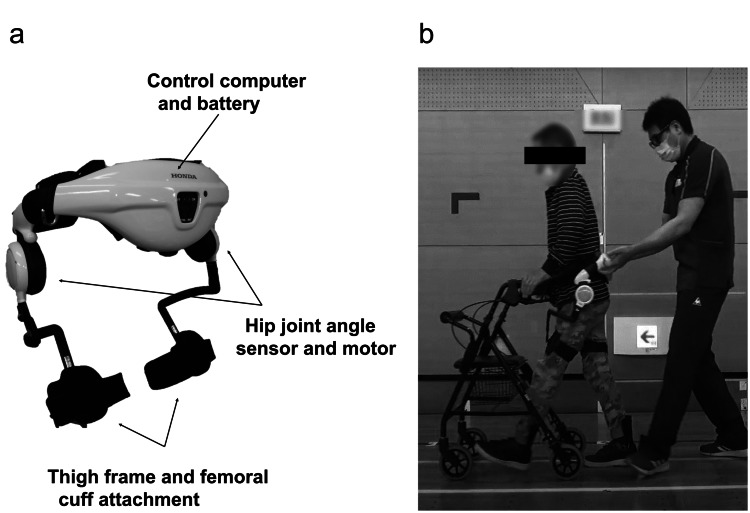
(a) Hip-wearable exoskeleton robot (HWA-01) and (b) gait training using HWA-01. HWA-01 can be used to assist hip joint flexion and extension during gait training. Information about the gait cycle obtained using the hip joint angle sensor was used to control the hip joint motor. Motor output was adjusted to elicit appropriate hip flexion and extension motions while walking in real time.

The patient’s gait improved until seven months after rehabilitation. However, no improvement in gait speed was observed for one month, and the improvement in gait ability from supervised to independent walking was not remarkable. On day 382 after disease onset, HGT was initiated (Figure 1A) with the aim of improving his gait and achieving independent walking (Figure 1B). Additionally, the HGT program was performed using a walker in a safe environment, considering the risk of falls.

This study was approved by the Ethics Committee of Ibaraki Prefectural University of Health Sciences (Approval No.: e204). Written informed consent was obtained from the patient for participation in and publication of this study.

Procedure

This was a single-case study based on the ABABA design. Phase A included conventional gait training, and phase B involved HGT during physical therapy. In phases A and B, physical therapy, including ROM exercises and ADL training (walking and stair climbing), was performed six days per week (five days on weekdays and one day on weekends). In phase A, the conventional gait program included level walking and treadmill gait training. The intervention time was 10-40 minutes per day. In phase B, the HGT program was performed five days per week, and the conventional gait program, similar to phase A, was performed one day per week. Additionally, the HGT program was only performed on weekdays from a management perspective. At the start of each HGT session, the physical therapist attached the HWA-01 to the patient and the torque settings from the previous session were applied. During HGT, the gait pattern was required to be as comfortable and close to a normal gait as possible with the assistance of the HWA-01. Additionally, the torque assistance was adjusted during the HGT session based on the patient’s feedback and the physical therapist’s observations. The physical therapist lightly held the HWA-01 to prevent falls and did not assist the patient with joint movements. The intervention time was 20-30 minutes per day. The patient used a dynamic plastic ankle foot orthosis for foot drop and walker as needed in training and ADL situations during phases A and B.

This study was conducted in the following sequence: phase A1 (three weeks), phase B1 (three weeks), phase A2 (four weeks), phase B2 (three weeks), and phase A3 (three weeks). During phases B1 and B2, the Borg scale [13] was measured to prevent overload during gait training. The Borg scale in the gait training was set to <14. The fitting with HWA-01 was checked for any adverse effects on skin conditions. During both phases, we evaluated the presence or degree of pain using a numerical rating scale (0-10 worst). Furthermore, a three-dimensional gait analysis was performed to assess the patient’s gait pattern at a comfortable walking speed (CWS) without HWA-01. The Noraxon MyoMotion system (Noraxon U.S.A., Inc., Scottsdale, AZ), which is an inertial measurement unit comprising an accelerometer, gyroscope, and magnetometer, was utilized for gait analysis. Data were recorded at a sampling frequency of 100 Hz.

Outcome measurements

Primary Outcome

The CWS, stride length, and cadence were measured as in our previous report [8] at the beginning of training in all phases.

Secondary Outcome

The secondary outcomes included GBS disability score, Medical Research Council (MRC) sum, six-minute walking test (6MWT), motor items of functional independence measure (motor-FIM), and Overall Neuropathy Limitations Scale (ONLS) scores. The secondary outcomes were collected at the beginning of the A1 phase and the end of each phase. Gait-related measurements, including CWS and 6MWT, were performed without HWA-01. The GBS disability score can classify the GBS severity on a seven-point scale (from 0 (healthy state) to 6 (dead)) based on muscle strength assessment in the upper and lower extremities [14]. The MRC is scored according to the sum of limb muscle strength ratings (0-60 points) on a six-point scale (from 0 (no visible/palpable contraction) to 5 (normal)) based on 12 muscles (right and left shoulder abduction, elbow flexion, wrist dorsiflexion, hip flexion, knee extension, and ankle dorsiflexion) [6]. The 6MWT scores are commonly used to assess an individual’s walking ability, particularly endurance [15]. The motor-FIM is a total score (13-91 points) of performance of self-care (six items), sphincter control (two items), transfers (three items), and locomotion (two items) and determines the degree of independence in performing ADLs [16]. ONLS is a neuropathy-specific upper and lower extremity activity measurement scale that utilizes a six-point scale (from 0 to 5 for the upper extremities) and an eight-point scale (from 0 to 7 for the lower extremities). The total score (0 (no disability) to 12 (maximum disability)) was used to evaluate neuropathy in the upper and lower limbs, as described previously [17].

Data analysis

The joint angles during walking at CWS were calculated using MR3 (Noraxon U.S.A., Inc.). The target joint angles included hip flexion-extension, knee flexion-extension, and ankle plantar flexion-dorsiflexion in the sagittal plane. Data analysis was performed based on 10 complete gait cycles. The changes in joint angles during walking at CWS in each phase were compared.

For the primary outcome analysis, we used Friedman’s repeated measures nonparametric test to compare the gait parameters (CWS, stride length, and cadence) in five phases. Multiple comparisons were conducted using the Wilcoxon signed-rank test, and p-values were adjusted using the Holm method. Statistical significance was set at 5% (*p* < 0.05). We performed all statistical analyses using R software version 4.3.2 (R Core Team, Vienna, Austria). Furthermore, we compared CWS changes in phases with minimal clinically important difference (MCID). The MCID of CWS in adults with pathology ranged from a small change of 0.10 m/s to a large change of 0.20 m/s [18].

We calculated the secondary outcomes to identify the differences between the phases. Furthermore, we compared the 6MWT changes in phases with the MCID. The MCID of 6MWT in patients with chronic inflammatory demyelinating polyradiculoneuropathy, a disease similar to GBS, has been reported to be 20 m [19].

Results

Intervention Time, Fatigue, and Pain

The average time to complete gait training in the conventional intervention phase was 22.7 minutes, whereas that in the HWA-01 intervention phase was 21.6 minutes. The average Borg scale scores during HGT were 13.07 ± 0.26 in the B1 phase and 12.67 ± 0.62 in the B2 phase. Fatigue and pain were not observed in phases A or B. No skin damage was induced by HWA-01.

Primary Outcomes

**Figure 2:**
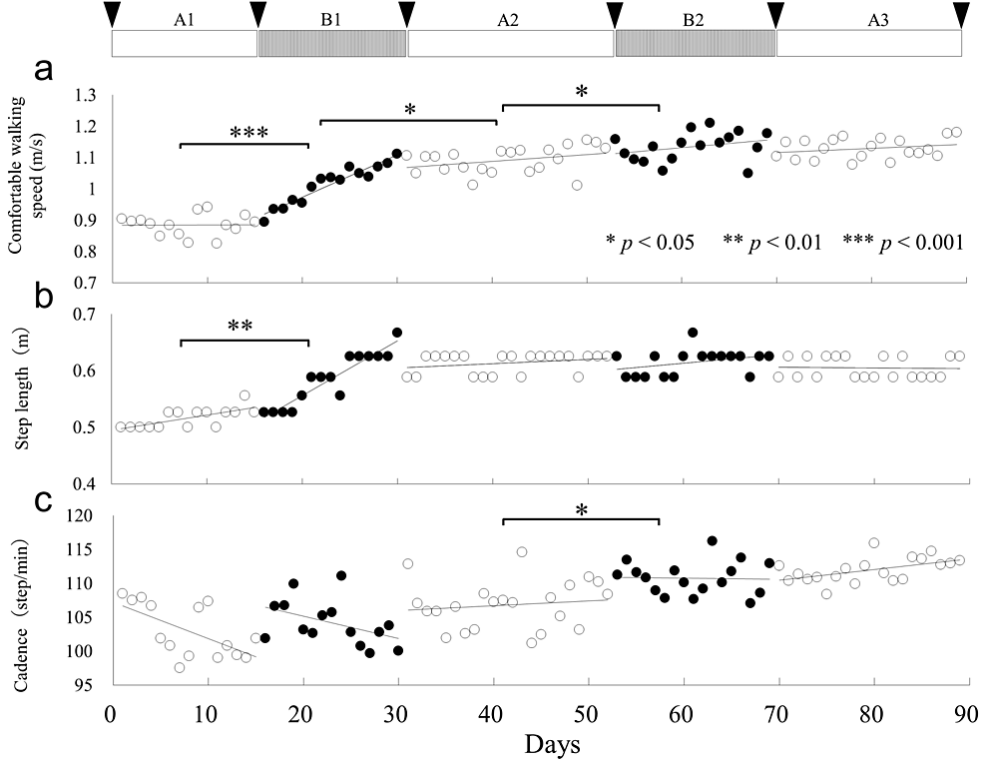
Intervention schedule and trends in comfortable walking speed (a), step length (b), and cadence (c) in each phase. The inverted triangles indicate the timing of the secondary outcome measurement. Open circles represent the conventional intervention phase (phase A), and closed circles represent gait training using the hip-wearable exoskeleton robot intervention phase (phase B).

Figure 2A shows the data regarding CWS. According to the Friedman test, the CWS significantly differed between the intervention phases (*χ^2^* = 44.16, *df* = 4, *p* < 0.001). Using the Holm method for multiple comparisons, a significant improvement was observed in walking speed from the A1 phase to the B2 phase over each adjacent phase (A1 vs. B1: *p* < 0.01; B1 vs. A2: *p* < 0.05; A2 vs. B2: *p* < 0.05). The CWS between the B2 and A3 phases did not significantly differ (*p* = 0.538). In contrast, in all nonadjacent phases, except for the B2 and A3 phases, significant differences were noted in terms of CWS (A1 vs. A2, B2, and A3: *p* < 0.01; B1 vs. B2 and A3: *p* < 0.01; and A2 vs. A3: *p* < 0.05). CWS improved from 0.89 m/s (A1 phase, before HGT) to 1.03 m/s (B1 phase, after HGT), which exceeds the MCID. Figure 2B shows data regarding the step length. Based on the Friedman test, the step length significantly differed between intervention phases (*χ^2^* = 37.606, *df* = 4, *p* < 0.001). The Holm method indicated a significant improvement in step length from the A1 phase to the B1 phase (*p* < 0.01); however, the step length did not differ in the other phases. Figure 2C presents the cadence data. Based on the Friedman test, a significant difference in cadence was observed between the intervention phases (*χ^2^* = 34.733, *df* = 4, *p* < 0.001). According to the Holm method, cadence significantly improved from the A2 phase to the B2 phase (*p* < 0.05). Regarding nonadjacent phases, cadence significantly increased in the A1 phase compared with that in B2 and A3 phases (all *p* < 0.01), in the B1 phase compared with that in B2 and A3 phase (all *p* < 0.01), and in the A2 phase compared with the A3 phase (*p* < 0.01).

Secondary Outcomes

Table 1 shows the MRC, 6MDT, motor-FIM, and ONLS scores, and Tables 2-4 show the change for MRC, motor-FIM, and ONLS scores in each phase. All items showed an improvement from the beginning of the A1 phase to the end of the A3 phase. In particular, the results of the 6MWT improved from the start to the end of the A1 phase, from the A1 phase to the B1 phase, and from the B1 phase to the A2 phase, which exceeds the MCID.

**Table 1:** Changes in Guillain-Barré syndrome disability score, muscle strength, exercise capacity, physical function, and activities of daily living score. Values in parentheses in the MRC sum score indicate the sum of the hip flexion, knee extension, and ankle dorsi flexion scores. Values in parentheses for 6MWT indicate the amount of change from the previous phase. GBS: Guillain-Barré syndrome; MRC score: Medical Research Council score for muscle strength; 6MWT: six-minute walk test; ONLS: Overall Neuropathy Limitations Scale; Motor-FIM: motor items of functional independence measure.

Outcomes	A1 start	A1 end	B1 end	A2 end	B2 end	A3 end
GBS disability score (0–6)	3	3	2	2	2	2
MRC sum score (0–60)	33 (18)	33 (18)	40 (19)	43 (22)	46 (23)	46 (23)
6MWT (m)	283.4	334 (+50.6)	373.6 (+39.6)	400.2 (+26.6)	385 (−15.2)	404.2 (+19.2)
ONLS score (0–12)	8	8	8	6	5	5
Motor-FIM (13–91)	38	40	46	51	59	60

**Table 2:** Change for each movement tested of the MRC score in each phase. R/L: right side/left side; MRC score: Medical Research Council score for muscle strength.

Movement tested (R/L)	A1 start	A1 end	B1 end	A2 end	B2 end	A3 end
Shoulder abduction	3/2	3/2	4/4	4/4	4/4	4/4
Forearm flexion	3/2	3/2	4/3	4/3	4/4	4/4
Wrist flexion	2/3	2/3	2/4	2/4	3/4	3/4
Hip flexion	4/4	4/4	4/4	5/4	5/5	5/5
Knee extension	4/4	4/4	5/4	5/4	5/4	5/4
Ankle dorsiflexion	1/1	1/1	1/1	2/2	2/2	2/2

**Table 3:** Change for each grade of the ONLS score in each phase. ONLS: Overall Neuropathy Limitations Scale.

Components	A1 start	A1 end	B1 end	A2 end	B2 end	A3 end
Arm grade	4	4	4	3	3	3
Leg grade	4	4	4	3	2	2
Total score	8	8	8	6	5	5

**Table 4:** Change for each item of the motor-FIM in each phase. Motor-FIM: motor items of functional independence measure.

Items	A1 start	A1 end	B1 end	A2 end	B2 end	A3 end
Eating	4	4	5	5	5	5
Grooming	1	1	1	2	2	3
Bathing	1	1	2	2	3	3
Dressing - upper body	1	1	1	1	1	1
Dressing - lower body	1	1	1	1	5	5
Toileting	2	2	3	3	4	4
Bladder management	6	6	6	6	6	6
Bowel management	6	6	6	6	6	6
Transfers bed/chair/wheelchair	5	5	5	6	6	6
Toilet	5	5	6	6	6	6
Tub/shower	1	3	4	4	4	4
Walk	1	1	1	4	6	6
Wheelchair	6	6	6	6	6	6
Stairs	4	4	5	5	5	5
Total motor-FIM (wheelchair)	43	45	51	53	59	60
Total motor-FIM (walk)	38	40	46	51	59	60

Changes in the Joint Angles During Gait

**Figure 3:**
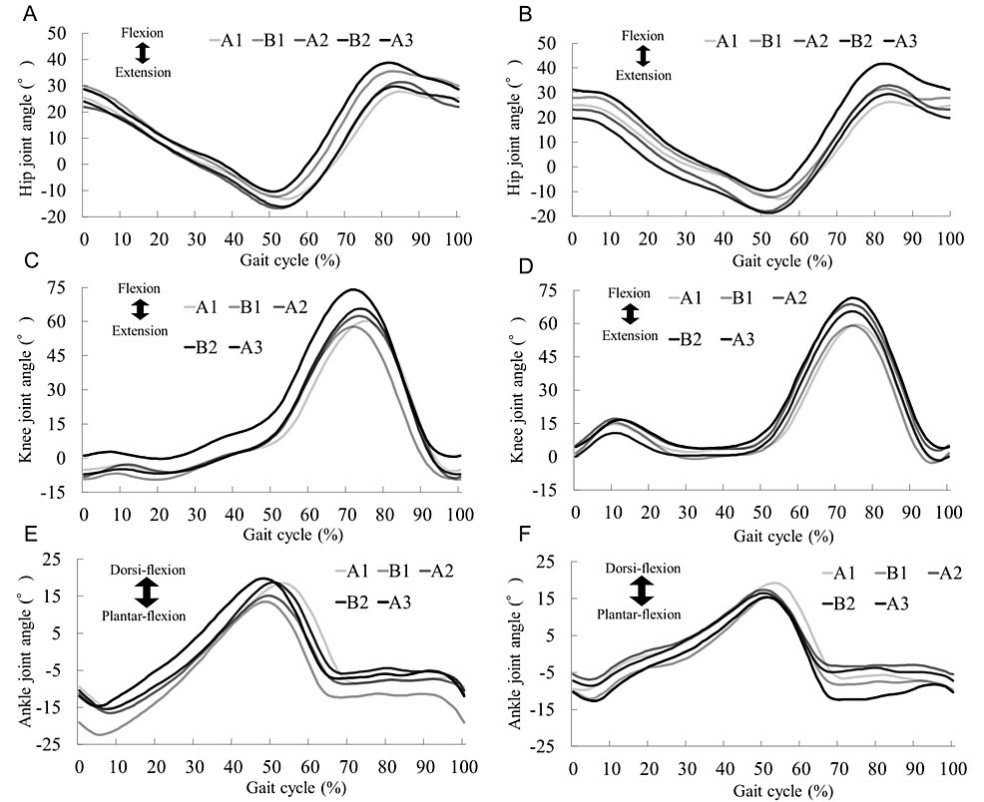
Average angular changes over a gait cycle in the hip, knee, and ankle joints recorded during the 10-m walking test at a comfortable walking speed. (A) Left hip joint, (B) right hip joint, (C) left knee joint, (D) right knee joint, (E) left ankle joint, and (F) right ankle joint. The darkness of the line indicates the intervention phase progression. The hip flexion angle increased on both sides after the B1 phase.

Figure 3 presents the joint angles during gait training obtained via three-dimensional gait analysis. The ROM during hip joint flexion-extension increased with the processing phases. In particular, the hip flexion angle increased from the A1 phase to the B1 phase. The knee joint flexion-extension angle revealed a trend toward knee hyperextension in the early left stance phase, which improved in the intervention phase. The ankle joint plantar flexion-dorsiflexion angle demonstrated a gait pattern similar to that of a normal gait throughout the intervention. Nonetheless, excessive plantar flexion of the ankle joint occurred due to knee hyperextension in the early left stance phase.

## Discussion

This report presents the case of a patient with GBS who showed improvement in gait ability after conventional rehabilitation combined with HGT. In this case, muscle strength (MRC score), walking endurance (6MWT), lower extremity function (ONLS score), and ADL (motor-FIM) improved, along with CWS, which was seen to be restored to the levels exhibited by healthy older people [20]. These findings suggest that high-frequency training combined with conventional physical therapy (e.g., ADL and ROM exercises) and HGT in this patient was effective. This patient's gait ability did not improve after one month of conventional gait training prior to phase A1. The results of the gait analysis at the start of the A1 phase suggest that the decrease in the forward and backward swing of the hip joint during gait led to a decrease in stride length and gait speed. The change in the hip joint angle may have dynamically influenced changes in the knee and ankle joints; however, we could not find any changes in the knee and ankle joints in this result.

HGT can provide symmetrical hip flexion and extension assistance based on motor output in the stance and swing phases, promoting the coordinated movement of both lower limbs [8,9]. HWA-01 has been used to treat several neurological diseases and is reported to improve gait speed [11] and symmetry [10,21] by increasing stride length. In our patient, HGT improved the hip angle during walking, and the increased stride length may have led to improved walking speed, similar to the results of previous studies. These improvements in gait speed and gait kinematics support our hypothesis that intervention at the hip joint in patients with GBS improves gait disturbance. Improvement in CWS after phase B2 was less than improvement in phase B1. In a study by Oberg et al., the mean walking speed in healthy older men aged 70-79 years was 1.18 ± 0.15 m/s [22]. The median CWS for this patient was 1.14 m/s in phase B2, indicating that the improvement in CWS had reached a level comparable to that of healthy older people.

The 6MWT score exceeded the MCID reported in a previous study [19], indicating that HGT improves energy efficiency [23]. A previous study showed that HGT reduced regional glucose metabolism in lower limb muscles in healthy older adults [24]. Therefore, the improvement in 6MWT from phase A1 to phase B1 might be influenced by the improved energy efficiency during gait with HWA-01. The patient was efficient during phase B1. The patient may have acquired an efficient gait in phase B1, and then training from phase B1 to phase A2 may have led to further 6MWT improvement. Furthermore, the upper and lower extremity function (ONLS) and muscle strength (MRC) scores reflected the effect of the rehabilitation programs throughout the entire period from phase A1 to phase A3. Motor-FIM was seen to exceed the MCID (22.0 points) reported in a previous study [25]. In this case, the improvement in motor-FIM included not only walking but also stairs, toileting, and dressing, indicating that, in addition to the effect of HGT, the long-term effect of ADL training from phase A1 to phase A3 contributed to improving the patient’s ADL. Thus, the improvement in ADL ability could be attributed to the implementation of ADL training in conjunction with improvements in upper and lower extremity function and muscle strength.

In the current case, the Borg scale results showed no exercise overload from HGT during phase B. Additionally, the intervention protocol allowed the safe implementation of HGT. As fatigue can significantly affect ADL capacity, social function, and the quality of life of patients with GBS [13], consideration of appropriate exercise load is essential. This report has some limitations. This was a single-case report. Although HGT was effective in improving functional mobility in patients with GBS, the results cannot be generalized. Furthermore, a more careful discussion is required to investigate whether the costs associated with implementing and using HWA-01 are acceptable in other common clinical settings. Future studies should aim to include larger sample sizes to confirm the efficacy of rehabilitation using HWA-01 training.

## Conclusions

Patients with GBS require long-term rehabilitation because of the prolonged motor recovery. In our patient with GBS, a combination of HGT and conventional rehabilitation helped improve gait ability, muscle strength, motor function, stride length with extending hip flexion angle, and cadence. HGT can potentially improve gait ability in patients with GBS who have residual gait disturbance. The HWA-01 is an easily transportable device that can be used after a brief explanation of its use. HGT should be preferred for patients with GBS, and its efficacy in reducing hospitalization days and promoting return to ADLs should be evaluated. However, further studies are needed to determine whether the costs of using the HWA-01 are feasible in other clinical settings.
